# Supplementary figures and images for: JMJ Histone Demethylases Balance H3K27me3 and H3K4me3 Levels at the HSP21 Locus during Heat Acclimation in Arabidopsis
Source: Biomolecules. 2021 Jun 7;11(6):852. doi: 10.3390/biom11060852 (PMC8227549; doi:10.3390/biom11060852)

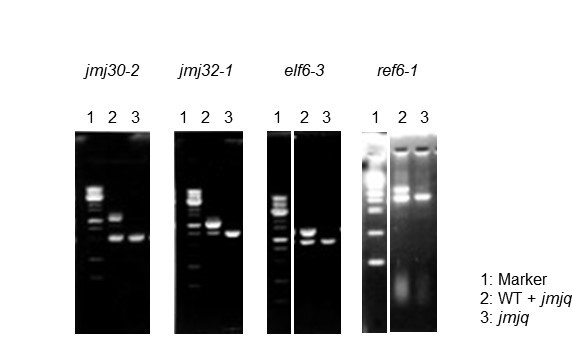

Supplement: Supplementary file 1 [file biomolecules-11-00852-s001.zip › biomolecules-1219279-supplementary.jpg]
